# Evaluation of Indigo carmine on hepatic ischemia and reperfusion injury^1^

**DOI:** 10.1590/s0102-865020200090000001

**Published:** 2020-09-23

**Authors:** Eduardo Alexandre Rancan, Eloísa Ianes Frota, Tábata Marina Nóbrega de Freitas, Maria Cecília Jordani, Paulo Roberto Barbosa Évora, Orlando Castro-e-Silva

**Affiliations:** I Graduate student, Faculdade de Medicina de Marília (FAMEMA), Marilia-SP, Brazil. Technical procedures; acquisition, analysis and interpretation of data, manuscript preparation.; II Master, Biochemistry, Division of Digestive Surgery, Department of Surgery and Anatomy, Faculdade de Medicina de Ribeirão Preto, Universidade de São Paulo (FMRP-USP), Ribeirao Preto-SP, Brazil. Acquisition and interpretation of data, statistics analysis.; III PhD, Full Professor, Division of Thoracic and Cardiovascular Surgery, Department of Surgery and Anatomy, FMRP-USP, Ribeirao Preto-SP, Brazil. Conception and design of the study, manuscript writing, critical revision.; IV PhD, Full Professor, Surgery and Anatomy Department, FMRP-USP, Ribeirao Preto-SP, Brazil. Conception and design of the study, analysis and interpretation of data, manuscript writing, critical revision.

**Keywords:** Ischemia, Reperfusion, Indigo carmine, Liver, Oxidative Stress, Mitochondria, Rats

## Abstract

**Purpose:**

To evaluate the effects of treatment with Indigo Carmine (IC) on rat livers subjected to ischemia-reperfusion injury.

**Methods:**

The animals were subdivided into 4 groups: 1.SHAM group(SH) - saline; 2.SHAM group with IC-2mg/Kg(SHIC); 3.IR group - rats submitted to ischemia and reperfusion with saline(IR); 4.IR group with IC-2mg/Kg(IRIC). The IR protocol consists of liver exposure and administration of drug or saline intravenously, followed by 60 minutes of ischemia and 15 of reperfusion. Liver samples were collected for biochemical analysis.

**Results:**

State 3 of mitochondrial respiration showed a significant worsening of the IRIC group in relation to all others. State 4 showed a difference between IRIC and SHIC. The Respiratory Control Ratio showed statistical decrease in IR and IRIC versus Sham. The osmotic swelling showed significant difference between SHxIR; SHICxIRIC and SHxIRIC. There was a significant increase in ALT in the IRIC group in relation to all the others. Concerning the nitrate dosage, there was a decrease in the group treated with IC(IRxIRIC). There was no difference regarding the dosage of Malondialdehyde.

**Conclusion:**

IC was not able to protect mitochondria from IR injury and proved to be a potentiating agent, acting in synergy with the IR injury promoting damage to the hepatocyte membranes.

## Introduction

When performing liver surgical procedures, such as resections and transplants, blood flow must be interrupted in order to contain bleeding. This control can be achieved through the Pringle Maneuver, which consists of a temporary occlusion of the portal triad composed of the hepatic artery, bile duct and portal vein^1,2^. In situations of circulatory shock, there is an important reduction in blood flow, which, as well as in surgical situations, leads to a state of ischemia and, therefore, injury to the liver parenchyma^1,3,4^. When the liver is revascularized, there is additional injury, characterizing the ischemia - reperfusion injury (IRI)^1^ and this is the main cause of post - surgical liver dysfunction^5^.

There are several mechanisms involved in IRI, including anaerobic metabolism, mitochondria disfunction, calcium overload, activation of Kupffer cells, oxidative stress, neutrophils, metalloproteinases, cytokines and chemokines^5^.

With oxygen depletion in the tissues, there is a change to anaerobic metabolism, as well as interruption of the electrons transport mechanisms of the respiratory chain, which causes a decrease in ATP levels, impairing the functioning of the Na^+^ / K^+^ ATPase pump and culminates in depolarization of the cell, along with calcium overload. Calcium accumulation is part of a metabolic pathway which triggers to activation of phospholipase C enzyme, protein kinase C, and also proteases, ATPase, reactive oxygen species (ROS) generation and, ultimately, hepatocytes apoptotic mechanisms^6^.

The reestablishment of blood flow, paradoxically, aggravates the injury started during ischemia. Due to previous environment created by ischemia, mainly, calcium overload, occurs an over production of ROS, whose mitochondria is a main source, mostly from the KC and neutrophils, along with production of inflammatory mediators^7^. Concomitantly, increased endothelial adherence, chemotaxis and even more injury from accumulation of free radicals feeds the inflammatory process previously established. Furthermore, a damaging environment produced by polymorphonuclear infiltration exerts an impairment in liver parenchyma and it is related to worsening organ function^5^.

Drugs that are able to improve microcirculation and also with free radicals scavenging properties, ergo compensating unbalance oxidative species production, have been tested and showed up as promising therapeutic targets for preventing IRI^4,8-10^.

Indigo Carmine or disodium salt of 5.5 indigo disulfonic acid, belongs to the indigoid family^11^. It has great applicability in diagnostic procedures and surgical evaluations using its intense color to assess the analyzed structure. It is a relatively safe drug due to its predominantly renal excretion^12-14^. In addition, it has pharmacological properties described, mainly in intravenous administration, with cardiovascular effects, such as reports of hypertensive peaks^15^, in addition to antioxidants^16^ and anti-mitotic properties^17^.

Meticulously, IC has a vasoconstrictor effect upon attack on two distinct fronts; the first, endothelium-independent, which suggests an action on smooth muscle with inhibition of guanylyl cyclase^18^, bringing it closer to the pharmacological properties of Blue of Methylene (MB)^19^, widely used in the treatment of vasoplegic syndrome^20^ and, recently, with potential application on IRI in the liver. The second and most auspicious pathway is endothelium-dependent, capable of preventing the formation of nitric oxide (NO), either by membrane receptor or calcium ionophore channel A23187^18^.

It is known that the control of calcium concentrations in the cytosol is critical, both in the production of NO by endothelial cells, which implies calcium increase for enzymatic process, and in the smooth muscle cell, where the relaxation of smooth muscle fiber occurs by calcium levels decrease^21,22^.

Furthermore, IC presents antioxidant properties related mainly to its scavenging of anion superoxide or oxygen singlet ability^23,24^ and, in view of this properties, has been used to detect ozone^25,26^. Moreover, it is a chelator of minerals such as copper, zinc, cobalt^27^ insofar as they participate in the Fenton reaction, which may contribute to avoid oxidative stress.

## Methods

The procedures with animals and the experimental protocols of this study were submitted to the Ethics Committee on Animal Experimentation (CETEA), FMRP-USP (protocol nº 014/2006).

Twenty four male Sprangue-Dawley rats between 200 - 400g were used. The animals were subdivided into 4 groups of 5-7 animals: 1. SHAM group (SH) with saline – rats that received saline solution intravenously and were subjected to anesthetic and surgical stress for 75 min without clamping the hepatic pedicle; 2. SHAM group with IC – 2mg/Kg (SHIC) - rats that received 2mg/Kg of intravenous IC and were subjected to anesthetic and surgical stress for 75 min without clamping the hepatic; 3. IR group with saline - rats that received saline solution intravenously and clamping of the hepatic pedicle for 60 min, followed by 15 min of reperfusion; 4. IR group with IC – 2mg/Kg (IRIC) - rats that received 2mg/Kg of intravenous IC and clamping of the hepatic pedicle for 60 min, followed by 15 min of reperfusion.

The animals were anesthetized with 0.2 mL/100g of a mixture containing 1mL of 2% xylazine and 1mL of 10% ketamine, applied to the right gluteal muscle. The rats were placed individually in a supine position on appropriate support with paws fixed in extension, followed by the abdominal region’s trichotomy. The surgical procedure was performed in a closed environment. Median laparotomy extended from the xiphoid process to the pubis was performed to expose the liver and gain access to the inferior vena cava (IVC). Saline solution or IC (2 mg/kg) was applied in intravenously, depending on the study group. Immediately afterward, it was made clamping of the hepatic pedicle with cerebral aneurysm clip in the designation Group I/R. This allowed the ischemia of the left medial and lateral lobes and right medial lobes for 60 minutes. The right lateral lobes and the caudate lobe were not subjected to ischemia. After the ischemic period, cerebral aneurysm clip was removed, beginning the period of reperfusion for 15 minutes. At the time of sacrifice, blood samples were taken and after clot retraction and centrifuged to obtain the serum for subsequent determination of alanine aminotransferase (ALT), aspartate aminotransferase (AST) and lactate dehydrogenase (LDH). Hepatic tissue samples were collected and processed immediately for assessing mitochondrial respiration parameters (O_2_ consumption rates in state 3 and 4 and respiratory control ratio - RCR), internal mitochondrial membrane permeability transition (mitochondrial swelling). Part of the tissue was frozen at -70°C for subsequent determination of the cellular oxidative stress (MDA) and determination of the NO.

### *Isolation of liver mitochondria*

Mitochondria were isolated by differential centrifugation. After the surgical procedure, the liver was removed immediately and placed in saline where it was washed. Then it was placed in medium containing 250 mM sucrose, 1 mM EGTA, and 10 mM Hepes-KOH, pH 7.2 in which it was perforated and homogenized in Potter-Elvehjem through 3 cycles of 15 seconds with a 1-minute interval. The homogenate was centrifuged at 770g for 5 minutes, and the resulting supernatant was centrifuged at 9800g for 10 minutes. The obtained pellet was suspended in 10 mL of medium containing 250 mM sucrose, 0.3 mM EGTA and 10 mM Hepes-KOH, pH 7.2, and centrifuged at 4500g for 15 minutes. The final pellet containing isolated mitochondria was suspended in 0.5 mL of medium containing 250 mM sucrose and 10 mM Hepes-KOH, pH 7.2. All mitochondria isolation steps were carried out at 4°C^28^.

### *Determination of mitochondrial protein*

The mitochondrial protein was determined using Comassie Plus (Bradford) Assay Kit *-* Thermoscientific at *595nm* in a Versamax microplate reader (Molecular Devices). The results obtained were expressed in mg / mL, using bovine serum albumin as a standard^29^.

### *Oxygen consumption by mitochondria*

Mitochondrial respiration was monitored in Hansatech-Oxygraph Plus *equipped* with an oxygen electrode. Mitochondria (1 mg/mL) energized with 5mM potassium succinate were added to the breathing medium containing 125 mM sucrose, 65 mM KC l, 1 mM KCl_2_, 2 mM KH_2_PO_4_, 0.1 mM EGTA and Hepes-KOH 10mM pH 7.4. At this point, electron transfer begins along the respiratory chain, generating consumption of O_2_, which is called mitochondrial respiration. State 4 respiration, also called basal respiration, consists of the consumption of oxygen dissociated from ATP synthesis. After the addition of 200 mM ADP, state 3 respiration begins, whose consumption of O_2_ by mitochondria is coupled to ATP synthesis by FoF1- ATPase^30^.

### *Determination of mitochondrial osmotic swelling*

Mitochondrial swelling is a method used to demonstrate the permeability transition of the internal mitochondrial membrane and was determined by decreasing the absorbance at 540 nm, using a Beckman DU 640B spectrophotometer (USA). Mitochondria energized with 5 mM potassium succinate were added to the medium containing 125 mM sucrose, 65 mM KCl, and 10 mM Hepes-KOH pH 7.4 and mediated by 20 µM CaCl_2_ and 1 mM KH_2_PO_4_; mitochondrial swelling was accompanied by a decrease in turbidity of the suspension, and consequently, a proportional decrease in absorbance^31^.

### *Determination of malondialdehyde (MDA)*

The malondialdehyde is one of the final products derived from the peroxidation of fatty acids, and its determination allows a convenient measure of lipid peroxidation. The colorimetric determination of MDA by its reaction with thiobarbituric acid was performed at 532 nm in a Versamax microplate reader (Molecular Devices) 1,1,3,3-tetramethoxypropane (0 to 100 µM) as standard. The results obtained were expressed in µM/mg of protein^32^.

### *Determination of nitrate (NO)*

Liver samples were collected at 4° and stored at -70°C. At the time of the test, the tissue was weighed, perforated, and homogenized at 10% (w/v) in 20 mM Tris HCl, pH 7.4, and the homogenate obtained was centrifuged at 5000 rpm for 10 minutes. The supernatant was collected for protein determination. The samples were then deproteinized by incubation with 95% ethanol for 30 minutes and centrifuged at 10.000 rpm for 5 minutes. With the supernatant obtained, NO 3 was measured by chemiluminescence NO / ozone. The sample (15 uL) was injected into the reaction chamber containing the reducing agent (0.8% vanadium chloride in 1 N HCl) at 80°C, which converts the nitrate into nitric oxide in equimolar amounts. Nitric oxide is dredged by nitrogen gas to the chemiluminescence chamber of the Sievers NO Analyzer (Sievers 280i NOA, Sievers, Boulder, CO, USA), where it reacts with ozone, emitting red light: NO + O_3_ → NO_2_ + O_2_; NO_2_ → NO_2_ + hv.

The photon emitted by the reaction was detected and converted into an electrical signal for the computer analysis. The area under the curve generated by the electric current corresponds to the concentration of nitric oxide in the sample. The nitrate concentration was calculated through a curve using sodium nitrate (100 to 1 µM) as a standard. The results obtained were expressed in uM/mg of protein^33^.

### *Determination of alanine aminotransferase (ALT), aspartate aminotransferase (AST) and lactate dehydrogenase (LDH)*

Serum enzyme measurements were performed using the kinetic method at 340 nm with the aid of the CELM SB-190 apparatus, using the Labtest kit^34^.

### *Statistical analysis*

Statistical analysis was performed using the GraphPad Prism program (GraphPad Software Corporation, version 8.0). The results were statistically analyzed using the Mann Whitney nonparametric test with significance level p<0.05 between groups.

## Results

**Figure 1:**
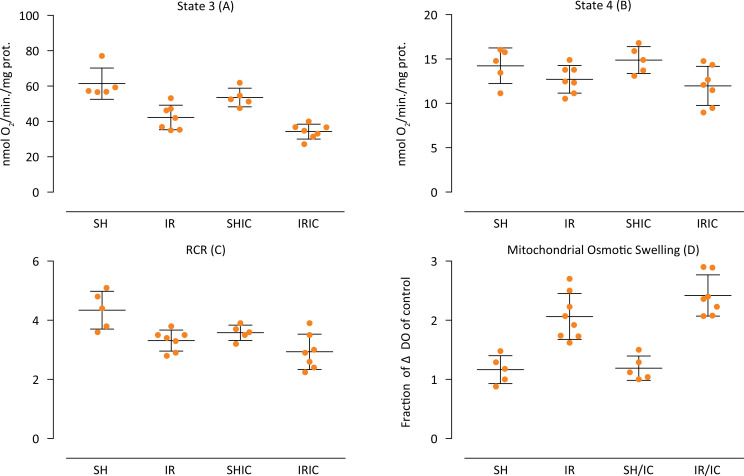
(A) The oxygen consumption by mitochondria in the presence of ADP – State 3, values expressed in nmol / min / mg (p<0.05: SH x IR; SHIC x IRIC; SH x IRIC; IR x IRIC). (B) The baseline oxygen consumption by mitochondria – State 4, values expressed in nmol / min / mg (p<0.05: SHIC x IRIC). (C) The State 3/ State 4 ratio, respiratory control ratio (RCR) (p<0.05: SH x IR; SH x IRIC). (D) Mitochondrial swelling induced by CaCl2 and KH2 PO4. p <0.05: SH x IR; SHIC x IRIC; SH x IRIC.

The rate of consumption of O_2_ in the presence of ADP (State 3) of all ischemic groups (IR, IRIC) was lower than the sham groups (SH, SHIC), demonstrating the effectiveness of the ischemic surgical procedure. The use of IC without performing surgical ischemia (SHIC) did not promote changes with statistically significant differences when compared to the Sham group (SH). The IRIC group worsened in State3 when compared to the IR group (Fig. 1A). The baseline mitochondrial consumption rate (State 4) evidences the integrity of mitochondrial membranes. IC when associated with ischemia (IRIC) showed difference statistically significant when compared to the SHIC group. The other groups were similar concerning State 4 (Fig. 1B). The Respiratory Control Ratio (RCR), given by the ratio between State 3 and State 4 is a useful parameter that relates directly proportional to the structural and functional integrity of the mitochondria. There was a statistical difference between the SH group and the IR and IRIC groups (SH x IR; SH x IRIC) (Fig. 1C). The mitochondrial osmotic swelling representing the transition of the mitochondrial internal membrane permeability, showed a significant difference between the SH x IR groups; SHIC x IRIC and SH x IRIC. It is known that ischemia leads to a loss of membrane selectivity, whereas the use of IC does not alter the IR lesion (Fig. 1D).

**Figure 2:**
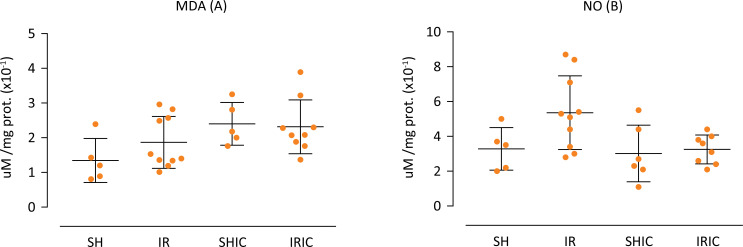
(A) MDA, there was no statistically significant difference between groups. (B) NO, p<0.05: SHIC x IR; IR x IRIC.

The determination of tissue MDA is a marker of lipid peroxidation and the researchers found no significant difference between groups (Fig. 2A). Regarding the determination of tissue NO, it presented a reduction between IRIC when compared with IR group (Fig. 2B).

**Figure 3:**
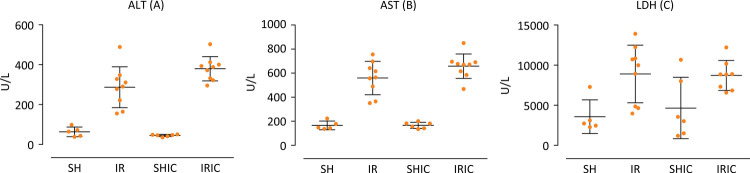
(A) ALT, p<0.05: SH x IR; SHIC x IRIC; SH x IRIC; IR x IRIC. (B) AST, p<0.05: SH x IR; SHIC x IRIC; SH x IRIC. (C) LDH, p <0.05: SH x IR; SH x IRIC; SHIC x IR.

In the measurement of serum concentration of alanine aminotransferase (ALT) there is a statistical difference between SH x IR, which guarantees sufficient ischemia; SH x IRIC, which shows that there is no protection by the drug against induced injury; IR x IRIC shows the worsening effect by Indigo Carmine in increasing the lesion IR (Fig. 3A). The serum determination of aspartate aminotransferase (AST) levels noted that the IC has no protective effect on the IRI since there is a significant difference between the groups SHIC x IRIC; SH x IRIC (Fig. 3B). The serum determination of lactate dehydrogenase (LDH) reveals a gap between SH x IRIC confirming the ineffectiveness of the drug in protecting the hepatocyte (Fig. 3C).

## Discussion

The results obtained show a successful experimental model of ischemia – reperfusion (IR) injury, that can be perceived by the differences between Sham and IR, with and without treatment.

Regarding the assessment of mitochondrial integrity, our results towards the consumption of O_2_ show that IC is nearly inert in effects on mitochondria when not subjected to the stress of ischemia and reperfusion, fact evidenced by the comparison between Sham groups with and without the drug in State 3 and State 4. However, considering the IRI, there is a synergism worsening the consumption of mitochondrial O_2_ when treated with IC. This is evident in State 3, which shows a more significant increase in IRIC group compared to all others; and in State 4, in which there is a difference (p <0.05) between treated Sham and IR (SHIC x IRIC), a fact that cannot even be seen between untreated Sham and IR, which, again, reinforces the drug enhancer effect in the face of IRI. These data indicate a possible interaction between the drug and the mitochondrial respiratory chain.

However, such consumption rates, when expressed in reason - RCR - are attenuated, what would probably be corrected with an increase in the number of animals in each group. The results found in the three parameters are similar to those obtained by Castro e Silva^35^in the treatment with Methylene Blue, which supports the pharmacological similarity of the compounds considered in this study^19^.

The synergism mentioned and suggested in this study, however, becomes less evident in the mitochondrial osmotic swelling, since there is a tendency of more severe injury of IRIC concerning IR, yet without a significant difference (p> 0.05). Whereas the treated Sham group (SHIC) is similar to the Sham group (SH), which again reinforces absolute inactivity of the drug in the absence of ischemia. It is well described in the literature that IRI may cause a transition of mitochondrial membrane permeability, leading to the so-called osmotic swelling^36^. Figure 1D shows that such mitochondrial phenomenon was present in both IR groups and that the treatment with IC was unable to protect the organelle membrane.

Mitochondria is a significant source of reactive oxygen species (ROS)^37,38^. Its massive production, mainly during reperfusion, leads to an imbalance known as oxidative stress, which is a fundamental part of IRI^39^. Figure 2B reveals that the treatment with IC was able to reduce the levels of oxidative stress to similar proportions as seen in Sham groups, highlighting a difference (p <0.05) between IR and IRIC groups. The antioxidant capacity of Indigo Carmine can be attributed to its double central bond, which, when cleaved, produces two molecules of sulfonic acid, constituting a reducing agent and superoxide anion scrubber^23^. However, our data could not endorse that, as seeing in Figure 2 in which MDA dosage showed no significant difference and NO dosage revealed (p<0.05) between SHIC x IR and IR x IRIC groups. Ergo, it is inconclusive if these results are either caused by inhibition of NO synthesis or its anti-oxidant properties.

Large amounts of NO can damage liver tissue and IC was capable of reducing NO levels similar to Sham groups as showed in Figure 2B. Nevertheless, our data suggest that these benefits were not capable to overcome the damage caused by the likely contribution in the imbalance between NO and endothelin-1, probably by inhibiting NO synthesis^18^. Such imbalance is a critical characteristic of IR injury^10^. Thus, the data from this study corroborate with Ramalho *et al*.^7^ about the importance of micro-circulatory insufficiency in IR injuries.

Along with oxidative stress, intracellular calcium homeostasis is one of the significant phenomena involved in IR^40^, since both are two vital pillars of injury^5,6^. Calcium overload is related to the excessive formation of ROS which can lead to organelle dysfunction and cell death^41^. Also, blocking calcium channels to avoid such overload proved to be able to mitigate the liver’s IRI^42^. It is described in the literature the ability to block the calcium ionophores A23187 by the IC, in addition to being able to inhibit the synthesis of endothelial NO through the activation of transmembrane receptors, whose activation cascades involve calcium^18,22,43^. Since IC acts on endothelial cells by a couple of ways, being them transmembrane receptors and calcium channel, that culminates in inhibition of NO production^18^. The enzyme NO–Synthase is present in mitochondria, and its output is controlled in typical situations^44^; however, when in excess, NO can lead to the formation of ONOO^-^ (peroxynitrite), which outcomes the transition of membrane permeability with a subsequent imbalance in calcium homeostasis^43^. Therefore, it would be expected that the lesion on mitochondria would be lessened when exposed to treatment.

Paradoxically, the high concentration of free calcium in the cytosol of endothelial cells is probably one of the main factors involved in the activation of the NOS enzyme (NO - Synthase) and this free radical is pointed out by many authors as a protector in IRI^21,45^. However, given the enzymatic variant iNOS (induced NO synthase) induced by inflammatory cytokines, there is an overproduction of NO^37^. This phenomenon is present in the IR lesion^37^ and in vasoplegic syndrome, a condition that post-transplant patients are more likely to develop^46^, and, for this, methylene blue and IC are therapeutic options^20^. The vasoconstrictor effect of IC in specific concentrations can be powerful enough to generate deleterious effects, as reported by Jeffords^15^ in one case report of hypertension blood malignant during cystography with use of IC. Hooson^47^, assessing IC toxicity, observed microinfarctions in rat livers submitted to 0.4% dietary doses, with significance (p <0.05) over control group.

The failure to achieve the expected protective effect of IC in this study permeates some possibilities. On the one hand, we may be faced with a misunderstanding in the results of intracellular calcium in an IR situation, as proposed by Duong^48^, when indicating that its overload in these injury models reduces the production of free radicals by mitochondria. On the other hand, we may have established here a particular hierarchy of importance in front of the performance of drugs that aim to mitigate the effects of IR. Thus, a therapy that could offer antioxidant effects and stabilize the membrane in terms of calcium influx without, however, harming the liver microcirculation is a promising option.

## Conclusions

The feasible antioxidant capacity of the IC was not enough to mitigate the hepatocyte injury. Furthermore, IC proved to be a potentiating agent, acting in synergy with the IR injury, promoting damage to the hepatocyte membranes.
